# Assessment of assertive community treatment in the Bizkaia Mental Health Services/Evaluación del tratamiento asertivo comunitario en la Red de Salud Mental de Bizkaia

**Published:** 2012-05-29

**Authors:** C. Pereira Rodriguez, J.J. Uriarte Uriarte, J. Moro Abascal, Fco. J. Martinez Corral

**Affiliations:** Director Manager of the Bizkaia Mental Health Services (RSMB, Red de Salud Mental de Bizkaia), Bilbao, Bizkaia, Spain; RSMB, Bilbao, Bizkaia, Spain; Officer for Alternatives to Hospitalisation, Margen Derecha, RSMB, Bilbao, Bizkaia, Spain; Officer for Management Control, RSMB, Bilbao, Bizkaia, Spain

**Keywords:** community mental health services, healthcare quality assessment, integrated delivery of healthcare, servicios comunitarios de salud mental, calidad de la atención de salud, prestación de atención de salud integrada

## Introduction

In recent decades, mental health services have improved with the development of healthcare models focused on community care, and balanced healthcare systems focused on the development of community services and the integration of hospital beds in general health facilities, such as general hospitals [1].

Assertive community treatment (ACT) teams were firstly adopted in the USA in the 1970s, with the explicit goal of providing very intensive community support for patients with severe conditions admitted in hospital units [2, 3]. Their main objectives are to maintain patients in contact with the health services, avoid treatment dropouts, improve community integration and avoid hospital admissions. ACT teams have become a standard model of very intensive community healthcare for people with severe mental illness in many countries with advanced mental health systems [4].

In Spain, ACT teams have been progressively implemented in several autonomous regions, led by the Asturias Mental Health Services more than 10 years ago. The so-called “Aviles model”, named after a town which has been a pioneer in the implementation of this type of service, has been recognised as an example of good practice in the Strategy for Mental Health of the Spanish National Health System.

In the Bizkaia Mental Health Services (RSMB), ACT teams started to be deployed in 2008, initially in only one health region, but by 2011 they had been developed to cover the whole of Biscay. The RSMB now has four ACT teams, one for each health region, the independent teams being composed of a nurse, an auxiliary nurse, a part-time psychiatrist and a part-time social worker. Currently, they provide care for 120 patients and are on duty from 09.30 to 16.30. During these hours, patients assigned to the programme have direct access to the healthcare team by mobile phone.

To be eligible to participate in this programme patients must meet the following criteria:

- Be aged between 18 and 65 years old
- Be diagnosed with a severe mental disorder (in particular, schizophrenia and affective psychosis)
- Have a history of poor adherence to standard community treatment
- Have a history of recurrent admissions and dropping out of treatment
- Have poor psychosocial functioning.

## Methods

The main objective of this study was to assess the impact of the establishment of a new healthcare service for patients with severe mental illness, in this case an ACT team, on the use of hospital facilities. Our hypothesis, endorsed by previous studies [5], is that patients receiving care by ACT teams have lower levels of admissions than similar patients under standard treatment. A secondary objective was to assess the impact of the ACT team on hospitalisation costs.

The activity of an ACT team was assessed from when it started in June 2008 in the Rehabilitation Unit of Zamudio Hospital. It cared for 57 patients between June 2008 and December 2010, carrying out 66 admissions and 36 discharges from hospital (Table 1).

We analysed the progression of the 33 patients involved in the programme over a period of two complete years (January 1st 2009 to 31st December 2010), and compared psychiatric admissions prior to and during the operation of the ACT programme. We considered the number of hospital stays in inpatient units before and after the programme, as well as the costs of hospitalisation and of ACT through the Rehabilitation Service.

## Results: (Table 2)

In the two years prior to their enrolment on the ACT programme, patients participating in the study occupied 5317 bed days, with an average stay of 197.31 days. After enrolling on the programme, hospital bed days decreased to 529 (–90%) with an average stay of just 6.89 days (–97%).

Healthcare costs associated with hospital stays of these patients in the two years prior to enrolling on the programme reached €1,630,192. In contrast, total healthcare costs for these patients in the two years since their enrolment on the ACT programme (costs of ACT+hospitalisation costs) were just €374,231, that is, a decrease of 77%.

## Discussion

The present study endorses numerous previous studies carried out in different contexts, confirming that ACT is effective in reducing the need for psychiatric hospitalisation in patients with severe mental disorders with low levels of adherence, complex healthcare needs and multiple admissions.

Most participants gained access to the ACT programme from inpatient units and day hospital services, often with a history of prolonged hospitalisations in previous years, and this may contribute to the spectacular results achieved. Further, we did not include in the study patients who were permanently withdrawn from the ACT programme, before the end of the 2 years of study, in some cases due to failure and readmission. It would be interesting to analyse the differential characteristics of this subgroup and try to identify the determinants associated with failure of their participation in the programme and monitoring by the teams.

In the present study, we did not collect data related to other clinical variables, such as improvement in symptoms, psychosocial functioning or social integration, quality of life or patient satisfaction with the service.

## Conclusion

Together with other alternatives to hospitalisation and residential social and healthcare facilities, our experience confirms that ACT programmes are an effective tool, for the development of a community model. In particular, these programmes (with their associated costs) are an efficient use of resources, leading to a significant decrease in the need for inpatient facilities.

Further research should investigate the impact of this type of service on other factors beyond hospitalisation, including patient clinical status, psychosocial functioning, quality of life and the satisfaction of patients and caregivers. It is also necessary to properly identify the type of patient who may benefit most from this type of service compared to standard care, in order to reduce redundancy and duplicity of services, while maintaining continuity of care.

## Conference abstract Spanish

## Introducción

En las últimas décadas, los servicios de salud mental han avanzado en el desarrollo de modelos asistenciales centrados en la atención comunitaria, y en sistemas equilibrados de atención centrados en el desarrollo de servicios comunitarios y de la integración de las camas hospitalarias en dispositivos sanitarios normalizados, como los hospitales generales [1].

Los Equipos de Tratamiento Asertivo Comunitario (ETAC) se desarrollaron en EEUU en los años 70, con el objetivo explícito de ofrecer soporte comunitario de muy alta intensidad para pacientes graves dados de alta de unidades hospitalarias [2, 3]. Su objetivo fundamental es el de mantener a los pacientes en contacto con los servicios, evitar abandonos de tratamiento, mejorar la integración comunitaria y evitar hospitalizaciones. Los ETAC se han impuesto como modelo estándar de asistencia comunitaria de alta intensidad para personas con enfermedad mental grave en buena parte de los países con sistemas sanitarios de atención a la salud mental avanzados [4]. En el estado español, los ETAC se han ido implantando de forma progresiva en diversas comunidades, implantación liderada por los Servicios Asturianos de Salud Mental desde hace más de diez años. El llamado “modelo Avilés”, localidad pionera en el desarrollo de este tipo de servicios, ha sido reconocido como ejemplo de buenas prácticas en la Estrategia en Salud Mental del Sistema Nacional de Salud.

En la Red de Salud Mental Bizkaia (RSMB), los ETAC iniciaron su despliegue en el año 2008, inicialmente en una única comarca asistencial, completando su desarrollo a todo el territorio en el año 2011. En el momento actual la RSMB dispone de cuatro ETAC, uno por comarca, con equipos autónomos compuestos por un Diplomado Universitario en Enfermería (DUE), un auxiliar de enfermería, un psiquiatra a tiempo parcial y un trabajador social a tiempo parcial. En la actualidad atienden a un total de 120 pacientes. El horario de atención es de 09.30 a 16.30. Durante el horario de atención los pacientes asignados al programa tienen acceso directo al equipo asistencial en un teléfono móvil.

Los pacientes susceptibles de ser integrados en este programa deben cumplir las siguientes características:

- Edad entre 18 y 65 años.
- Diagnóstico de Trastorno Mental Grave, fundamentalmente psicosis esquizofrénicas y afectivas.
- Antecedentes de mala adherencia al tratamiento comunitario estándar.
- Hospitalizaciones repetidas, abandonos reiterados del tratamiento.
- Funcionamiento psicosocial precario.

## Metodología

El presente estudio tiene como objetivo evaluar el impacto de la puesta en marcha de un nuevo servicio asistencial para pacientes que padecen trastorno mental grave, en este caso un ETAC, en el uso por parte de los pacientes asignados de recursos de hospitalización. La hipótesis, refrendada por estudios previos [5], es que los pacientes que reciben atención por parte de ETAC consumen menos días de hospitalización que los que reciben atención estándar. De forma secundaria se analiza la repercusión del equipo de ETAC en los costes de hospitalización.

Se analizó la actividad asistencial de un ETAC que inició su actividad en junio de 2008 desde la Unidad de Rehabilitación del Hospital de Zamudio. Entre junio de 2008 y diciembre de 2010 atendió a 57 pacientes distintos, realizó 66 admisiones y 36 altas (Tabla 3).

Se ha analizado en el estudio la evolución de los 33 pacientes que permanecen en el programa durante los dos años del estudio (del 1 enero 2009 al 31 diciembre 2010), y comparado el uso de hospitalizaciones psiquiátricas en los dos años previos con el de los dos años de permanencia en el ETAC. Se toman como referencia las estancias hospitalarias anteriores y posteriores causadas en unidades de hospitalización y los costes de hospitalización y TAC del Servicio Rehabilitación.

## Resultados

Los pacientes incluidos en el estudio utilizaron, en los dos años previos a su inclusión en el ETAC, 5.317 estancias en hospitalización con una estancia media de 197,31 días. Desde su admisión al ETAC, las estancias hospitalarias descendieron a 529 (–90%) con una estancia media de 6,89 días (–97%) (Tabla 4).

Los costes asistenciales por estancias hospitalarias originados por los pacientes incluidos en estudio en los dos años previos a la inclusión en el ETAC alcanzaron los 1.630.192€. Los costes asistenciales totales originados por los pacientes desde su ingreso en el ETAC (coste ETAC+coste hospitalización) han supuesto 374.231€, es decir un 77% inferiores.

## Discusión

El presente trabajo refrenda múltiples estudios previos realizados en distintos contextos, confirmando que los ETAC son eficaces para reducir las necesidades de hospitalización psiquiátrica en pacientes con Trastorno Mental Grave (TMG) con baja adherencia, necesidades asistenciales complejas y múltiples hospitalizaciones.

En el presente estudio se han incluido pacientes que mayoritariamente accedieron al ETAC desde unidades hospitalarias y servicios de hospital de día, a menudo con hospitalizaciones prolongadas en los años previos, circunstancia que probablemente contribuye a la espectacularidad de los resultados. Tampoco se han incluido en el estudio aquellos pacientes que fueron dados de alta del ETAC de forma definitiva antes de los dos años del periodo estudiado, en ocasiones por fracaso y rehospitalización. Sería de interés analizar las características diferenciales de esta población y los condicionantes asociados al fracaso en la integración y seguimiento por parte de estos equipos.

En el presente estudio no se han recogido datos relativos a otras variables clínicas, como mejoría en lo síntomas, en el funcionamiento psicosocial e integración social, calidad de vida o satisfacción con el servicio.

## Conclusión

EL ETAC es un dispositivo eficaz, junto con otras alternativas a la hospitalización y dispositivos residenciales socio-sanitarios, para el desarrollo del modelo comunitario siendo además sus costes una demostración de uso eficiente de los recursos y con una reducción significativa de las necesidades de hospitalización.

Es necesario conocer el impacto de este tipo de servicio sobre otros factores más allá de la hospitalización, incluyendo el estado clínico, el funcionamiento psicosocial, la calidad de vida y la satisfacción de los pacientes y sus cuidadores. Es necesario asimismo delimitar bien el tipo de paciente que se beneficia de este tipo de servicios en contraposición con la atención estándar, con el objetivo de no mantener servicios redundantes o que solapen su actividad, asegurando la continuidad asistencial.

## Figures and Tables

**Table 1.**
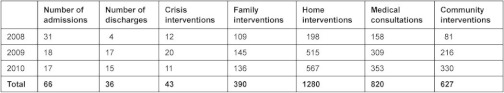
Care activity of the ACT programme. June 2008–December 2010

**Table 2.**
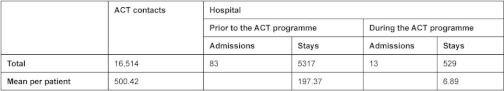
Hospital stays of patients participating in the ACT programme

**Tabla 3.**
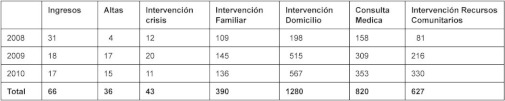
Actividad Asistencial ETAC, Junio 2008–Diciembre 2010

**Tabla 4.**
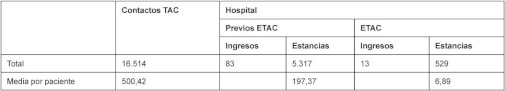
Estancias hospitalarias pacientes ETAC
